# Application of two machine learning algorithms to genetic association studies in the presence of covariates

**DOI:** 10.1186/1471-2156-9-71

**Published:** 2008-11-14

**Authors:** Bareng AS Nonyane, Andrea S Foulkes

**Affiliations:** 1 Division of Biostatistics and Epidemiology, School of Public Health and Health Sciences, University of Massachusetts Amherst, MA, USA

## Abstract

**Background:**

Population-based investigations aimed at uncovering genotype-trait associations often involve high-dimensional genetic polymorphism data as well as information on multiple environmental and clinical parameters. Machine learning (ML) algorithms offer a straightforward analytic approach for selecting subsets of these inputs that are most predictive of a pre-defined trait. The performance of these algorithms, however, in the presence of covariates is not well characterized.

**Methods and Results:**

In this manuscript, we investigate two approaches: Random Forests (RFs) and Multivariate Adaptive Regression Splines (MARS). Through multiple simulation studies, the performance under several underlying models is evaluated. An application to a cohort of HIV-1 infected individuals receiving anti-retroviral therapies is also provided.

**Conclusion:**

Consistent with more traditional regression modeling theory, our findings highlight the importance of considering the nature of underlying gene-covariate-trait relationships before applying ML algorithms, particularly when there is potential confounding or effect mediation.

## Background

The primary aim of many population-based genetic association studies is to characterize the relationship among multiple single nucleotide polymorphisms (SNPs) and a continuous or binary trait. Crucial to this endeavor is consideration of environmental, demographic and clinical factors that may themselves be associated with one another, the genotype and/or the trait under consideration. In this manuscript we consider several different approaches to handling covariates in the context of applying two machine learning (ML) algorithms, random forests (RFs) and multivariate adaptive regression splines (MARS). Specific consideration is given to underlying models of association and sensitivity of each approach for detecting true effects. Measures of false discovery are also reported.

RFs is a non-parametric approach, originally proposed by Breiman, L. (2001) [1], and represents an extension of classification and regression trees (CART) of Breiman, L. et al. (1984) [2]. MARS [3] is a related procedure that is theoretically more amenable than CART to underlying additive structure, as noted by Hastie, T. et al. (2001) [4]. Notably, several other ML learning algorithms have been described, including: support vector machines [5,6], neural networks [7], Bayesian variable selection [8,9] and k-nearest neighbor method [4,10]. We focus on RFs and MARS since they are being increasingly applied to population-based genetic association studies and may be preferable in the case of large datasets with missing values, mixed variable types and some irrelevant predictors [4].

Studies of the performance of ML algorithms include investigations of additive effects, statistical interaction, correlation among predictors, false positive rates and instability resulting from slight perturbations of the data [4,11-13]. Two recent review articles describe several applications of ML algorithms to find genotype-trait associations in the presence of covariates [14,15]; however, within each of the applications presented, a single approach to handling covariates is described. Typically, genetic indicators and environmental factors are entered into the ML algorithm as potential predictors. Stratified and matched-pair analysis to account for potential confounders are also described [16,17]. Taioli, E. and Garte, S. (2002) [18] highlight the importance of considering the nature of gene-trait confounding in the context of metabolic genes. Several recent manuscripts describe, for the general regression setting, the potential error associated with treating covariates as confounders when in fact they are effect mediators [19-22]. Finally, a comparative analysis of machine learning methods and logistic regression in the context of association studies has been presented recently [23]. To our knowledge, the existing literature does not include a systematic overview of the relative performance of ML algorithms across the many different approaches for handling covariates and underlying models of association. The present manuscript characterizes this for the application of two ML algorithms to population-based genetic association studies.

We present results of a simulation study aimed at characterizing the comparative performance and interpretation of several approaches to handling covariates. Specifically, we consider four strategies for handling covariates in the context of uncovering genotype-trait associations: (1) including covariates as predictors; (2) stratifying by covariates; (3) residualizing the outcome by covariates; and (4) ignoring the covariates. Several underlying models are also considered, namely additive models with and without predictors that are themselves associated, models with statistical interaction (i.e. effect modifiers) with and without main effects, and models of conditional association.

We begin by briefly outlining our notation. Let **Y **= (*y*_1_, *y*_2_,....*y*_*n*_)^*T *^be the vector of responses where *n *is the number of individuals in our sample and suppose **X **is an *n *× *p *matrix of genotype (SNP) variables with *ith *row given by **X**_*i *_= (*x*_*i*1_, *x*_*i*2_,..., *x*_*ip*_) and corresponding to individual *i*, *i *= 1,..., *n*. Further suppose **Z **is an *n *× *q *matrix with *ith *row given by **Z**_*i *_= (*z*_*i*1_, *z*_*i*2_,..., *z*_*iq*_) corresponding to the vector of covariate values for individual *i*. We assume throughout this manuscript that primary interest lies in correctly identifying associations between **Y **and **X **while accounting for **Z**.

### Random forests

Both classification and regression trees (CARTs) involve recursively partitioning individuals in a tree-like structure. The root node, consisting of all individuals in a sample, is split based on the value of one of the predictor variables into what are called left and right daughter nodes. Selection of the best predictor variable at each node is based on minimizing the within-node impurity (prediction error) in the resulting daughter nodes. In a classification tree for a categorical **Y**, node impurity is typically measured by the Gini index while for a regression tree on a continuous **Y **impurity is often measured by the mean square error. Splitting continues until a pre-specified criterion is reached, usually a minimum number of observations in terminal nodes. Finally, to obtain optimal trees that balance complexity and predictive power, some nodes may be removed in a pruning process [2].

A random forest (RF) is comprised of an ensemble of trees, each obtained via a recursive partitioning algorithm, as described above. The trees of a random forest are built such that at each node, a new random subset of all predictors is drawn and from this, a splitting variable is selected. This has the advantage of appropriately handling correlated predictors and avoids over-fitting. A measure of variable importance is obtained for each predictor based on all of the trees in the forest. Typically, the rank order of the importance score is reported [11,24,25]. A complete discussion of the RF methodology can be found in Breiman, L. (2001) [1] and straightforward implementation is achieved using the R package randomForest.

### Multivariate adaptive regression splines

Multivariate adaptive regression splines (MARS) is an alternative ML approach that involves fitting a series of reflective basis functions and their products. A complete discussion of model fitting and pruning is provided in Friedman, J. (1991) [3] and Hastie, T. et al. (2001) [4]. In this study, for ease of presentation, we consider a dominant genetic model so that genotype predictors in the form of SNPs are coded as indicators for the presence of at least one variant allele. Other genetic models such as additive, recessive and co-dominant are reasonable and both MARS and RF can be applied to these alternative settings. Treating SNPs as 3-level factor variables has the advantage of not requiring a prior knowledge of the underlying genetic model. In this case, we can generate two binary variables for each SNP and proceed in the same manner as described below. Interestingly, in the case of binary predictor variables, MARS is closely related to CART as we now illustrate.

Suppose we are interested in the set of predictors given by *X*_1_,..., *X*_*p*_. As described in Hastie, T. et al. (2001) [4], MARS begins by considering the model given by:

(1) *Y *= *α*_0 _+ *α*_1_(*X*_*j *_- *t*)_+ _+ *α*_2_(*t *- *X*_*j*_)_+ _+ *ϵ*;

where *t *∈ {*x*_*j*_} for each *j *= 1,..., *p*. Here *ϵ *is measurement error, *t *is an element of the set of observed values *x*_*j *_of the predictor *X*_*j*_, and (·)_+ _denotes the positive component of the argument within the parentheses. In the setting of binary predictors, we have that *t *∈ {0, 1} and thus Equation 1 reduces to:

(2) *Y *= *β*_0 _+ *β*_1 _*X*_*j *_+ *ϵ*.

The best predictor $X_{j}^{*}$ is defined as the variable that leads to the greatest reduction in the residual sums of squares. Notably, this first step is identical to the regression tree approach described above. In MARS, a model set ℳ is then defined as the set of functions (in our case predictors) given by {1, *X*_*j*_}. The next step is to fit models that involve products of the elements of ℳ and the predictor variables. That is, models of the form:

(3) $$Y=\beta_{0}+\beta_{1}X_{j}^{*}+\beta_{2}X_{k}+\epsilon$$

and

(4) $$Y=\beta_{0}+\beta_{1}X_{j}^{*}+\beta_{2}X_{j}^{*}X_{k}+\epsilon$$

are considered for *k *= 1,..., *p*. The difference between CART and MARS becomes apparent at this stage since CART does not allow for models of the form given by Equation 3. That is, MARS is more conducive to modeling additive structure across predictors. Again the best predictor is chosen, this time of the form *X*_*k *_or $X_{j}^{*}$*X*_*k*_, and then added to the model set ℳ. The process is repeated recursively to build a model of both additive and interaction terms. Finally, a backward deletion procedure is applied to reduce overfitting. MARS is also straightforward to implement using the R packages Earth and Mars.

## Methods

In this section, we begin by describing several simple models of association for genotypes, covariates and a single quantitative trait. We then describe a few reasonable approaches to handling covariates. Finally, a description of the simulation approach is provided.

### Models of association

Consideration is given to 6 underlying models of association involving genotypes (**X**_*i*_), covariates (**Z**_*i*_) and the dependent continuous trait (*Y*_*i*_) where *i *= 1...*n *indicates individuals. Notably **X**_*i *_and **Z**_*i *_can each be scalars or vectors, depending on the number of genotypes and covariates under investigation. In all cases, the errors denoted *ϵ*_*i *_are assumed to be independent and normally distributed with mean 0 and variance *σ*^2^. The following models of association are considered.

MODEL 1: ADDITIVE. An additive model is given by:

(5) $$Y_{i}=X_{i}^{T}\beta +Z_{i}^{T}\gamma +\epsilon_{i}$$

In this model, **X**_*i *_and **Z**_*i *_are additive with effects on the trait *Y *equal to ***β ***and ***γ ***respectively.

MODEL 2: ADDITIVE WITH CONFOUNDING. An additive model with confounding is again described by Equation 5. In this case, however, the variables **X **and **Z **are assumed to be correlated with one another.

MODEL 3: ADDITIVE WITH EFFECT MEDIATOR. An effect mediator is defined as a variable that is within the causal pathway to disease. For the third model of association, we assume that the covariate **Z **is in the causal pathway between the genotype **X **and the trait *Y *. This is described formally by the following two models:

(6) $$Z_{i}=X_{i}^{T}\beta +\epsilon_{i}$$

and

(7) $$Y_{i}=Z_{i}^{T}\gamma +\epsilon_{i}^{*}$$

MODEL 4: INTERACTION. A model for statistical interaction between the genotype (**X**) and the covariate (**Z**) has the form:

(8) $$Y_{i}=X_{i}^{T}\beta +Z_{i}^{T}\gamma +{\left(XZ\right)}_{i}^{T}\zeta +\epsilon_{i}.$$

Here (**XZ**) is used generically to denote one or more multiplicative terms involving both *X *and *Z*. The model implies that the effect of the combination of **X **and **Z**, given by ***ζ ***is greater than the sum of the effects of **X **and **Z **alone.

MODEL 5: INTERACTION WITHOUT MAIN EFFECTS. A model for interaction without main effects is given by:

(9) $$Y_{i}={\left(XZ\right)}_{i}^{T}\zeta +\epsilon_{i}$$

This model assumes that there is no effect of **X **or **Z **unless both **X **and **Z **are present.

MODEL 6: CONDITIONAL ASSOCIATION. Finally, a model for conditional association is given by:

(10) $$Y_{i}=Z_{i}^{T}\beta +{\left(XZ\right)}_{i}^{T}\zeta +\epsilon_{i}$$

This model implies that the genotype **X **affects the trait *Y *only in the presence of the covariate **Z**, that is for **Z **> 0. The strength of the relationship between *Y *and the predictor variables in these models is measured by the effect size, which is defined as the expected value of the regression coefficient *β *minus the hypothesized value, given by 0 in our setting, and divided by the common standard deviation [26].

### Approaches to handling covariates

We consider 4 approaches to handling covariates in the application of a machine learning algorithm. Each of these has been described previously in the literature.

APPROACH 1: INCLUDE AS PREDICTOR. One approach to handling covariates is to include them as potential predictors in the machine learning algorithm. That is, define the set of potential predictor variables by (*X*_1_,..., *X*_*p*_, *Z*_1_,..., *Z*_*q*_).

APPROACH 2: STRATIFY. A second approach is to first stratify our sample by the value(s) of one or more covariates. For example, suppose *Z *is an indicator variable for smoking status. A stratified analysis involves first organizing our data according to the level of *Z *so that smokers (*Z *= 1) are in one group and non-smokers (*Z *= 0) are in another group. Next, a machine learning algorithm can be applied to both subgroups separately to determine the *X *variables that are most predictive within smokers and non-smokers respectively.

APPROACH 3: RESIDUALIZE. A third approach is to first regress the outcome *Y *on the covariates *Z *by fitting a linear model of the form:

(11) *Y*_*i *_= *α *+ *Z*_*i *_*β *+ *ϵ*_*i*_

A new *residualized Y*_*i *_given by ${\tilde{Y}}_{i}$:

(12) $${\tilde{Y}}_{i}=Y_{i}-{\hat{Y}}_{i}=Y_{i}-(\hat{\alpha }+Z_{i}\hat{\beta })$$

is calculated based on a model fitting procedure where $\hat{\alpha }$ and $\hat{\beta }$ are least squares estimates. The machine learning algorithm is then applied to this new outcome, $\tilde{Y}$ with potential predictors, *X*_1_,..., *X*_*p*_.

APPROACH 4: IGNORE Finally, application of a machine learning algorithm without regard to covariates is considered. In this case, only the set of predictors *X*_1_,..., *X*_*p *_(and not *Z*_1_,..., *Z*_*q*_) is used in the analysis.

### Simulation approach

For our simulation study, we begin by generating 8 binary genotype indicators, (*X*_*j *_: *j *= 1,..., 8) and a single covariate (*Z*). For example, *X*_*j *_may be an indicator for the presence of at least one variant allele at a given locus or across multiple loci within a gene and *Z *may be an indicator for smoking status. Both binary and continuous covariates are considered. Genotype frequencies of 0.50 and 0.25 are assumed. This corresponds to variant allele frequencies of 0.293 = 1 - $\sqrt{1-0.5}$ and 0.134 = 1 - $\sqrt{1-0.25}$, respectively. The trait *Y *is generated under each of the 6 models described in Section. In each case a single genotype predictor is assumed to be associated with the trait and without loss of generality we let this be *X*_1_. We assume a range of effect sizes for this predictive genotype and the interaction terms involving it, while the covariate effect size is fixed at 0.5. That is, we vary values of *β*/*σ *and *ζ*/*σ*. In cases where the underlying model has an interaction term, the Earth function is run with the option *degree = 2 *which allows for two-way interaction terms in the MARS model. Samples of size *n *= 500 are generated in each of *S *= 1000 simulations per model and condition.

For MODEL 2, we use the method of Kang, S. and Jung, S. (2001) [27] to generate correlated binary variables. Values of the correlation between *X *and *Z *ranging from 0.2 to 0.9 are considered. In the case of a continuous covariate, the discrete approach described by Tannenbaum, S. et al. (2001) [28] is applied to induce correlation between *X *and *Z*. For MODEL 3, we simulate a mediator binary variable *Z *via a logistic regression on *X *and let *Z *= 1 if *ψ *≥ $\hat{p}$ or *Z *= 0 if *ψ *<$\hat{p}$ where *ψ *is a uniform random number between 0 and 1 and $\hat{p}$ is a predicted probability that *Z *= 1 conditional on X. In this case we let the effect size of X (on Z) vary and set the effect of Z (on Y) to equal 2. When *Z *is a continuous mediator variable, it is simulated by a simple linear regression on *X *with normal errors with mean 0 and variance equal to 1. In all other models, a continuous *Z *is simulated from a normal distribution with mean 0 and variance 1, and a binary *Z *is simulated with a frequency of 0.5.

For the RF setting, the variable importance score is recorded for all inputs. In the MARS setting, the set of terms selected in the final model is recorded. We focus on the true and false discovery rates for the genotype *X*_1 _in the presence of a covariate. In the context of RFs, we define the true discovery rate (TDR) as the proportion of simulations in which the truly predictive genotype *X*_1 _is ranked highest or second highest to *Z*. Under MARS, TDR is defined as the proportion of simulations in which *X*_1 _is in the final model set and is selected first, second to *Z *or in interaction with *Z*. While similar to power, the term TDR is used since formal testing is not performed. This term is intended to reflect the exploratory nature of machine learning algorithms. False discovery rate (FDR) is defined, under the null model of no association of *X *or *Z *with *Y*, as the maximum proportion of simulations in which any of the inputs has the highest importance score given by RF or is selected first in the final model set of MARS.

## Results

### Simulation study

**Figure 1:**
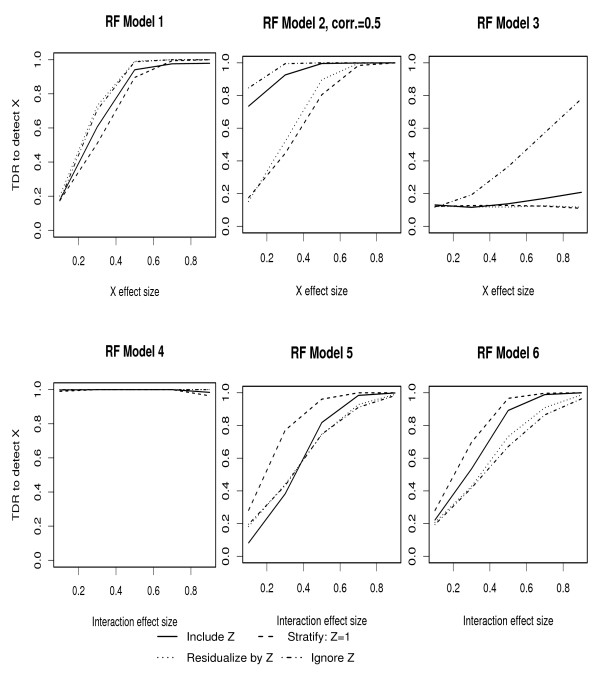
TDR to detect genotype effects under all approaches to handling covariates using Random Forests: For all models *Z *effect is fixed at 0.5 except for Model 3 where *Z *= 2. For Model 2, corr(*X*_1_, *Z*) = 0.5. For Models 1–3 *X*_1 _effect is varied over [0.1,0.9] while for Models 4–6 the effect sizes of main effects, if a model has main effects, are fixed at 0.5 and the effect size of the interaction term is varied over [0.1,0.9].

**Figure 2:**
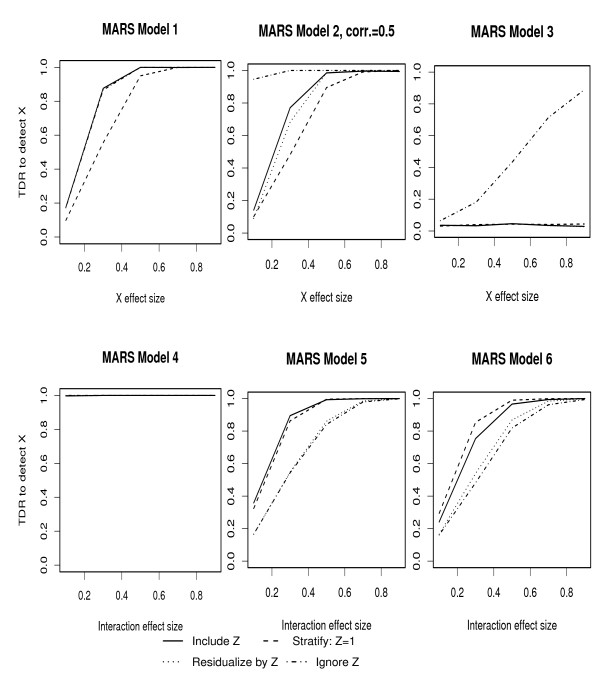
TDR to detect genotype effects under all approaches to handling covariates using MARS: *Z *and *X*_1 _effect sizes are as in Figure 1.

The plots of changes in TDR as a function of the change in effect size of *X*_1 _or (*X*_1_, *Z*) interaction are given in Figures 1 and 2, for RF and MARS respectively, and for each of the models described above. These results are obtained based on a binary covariate and genotype frequencies of 0.5. A summary of these figures is given in Table 1. In most cases, RF and ML have reasonable TDR to detect moderate genotype effect sizes (≥ 0.5), with the notable exception of Model 3. Under Model 3 in which the covariate (*Z*) is in a causal pathway between the genotype (*X*_1_) and outcome (*Y*), reasonable discovery rate is only achieved by ignoring *Z *and for relatively high effect sizes. As expected, Model 4 which has main effects and interaction terms, shows high TDR at very low effect sizes for all approaches to handling the covariate. Under Models 5 and 6 in which the effect of X on Y is only through an interaction with *Z*, RF appears to have slightly lower discovery rate than MARS at moderate effect sizes when using the residualize and ignore approaches. When applying these ML algorithms under the null model of no *X *or *Z *association with *Y*, we obtain FDR of 13% for RF and 4% for MARS.

**Table 1:** Summary of TDR analysis for binary genotypes and covariates

	Approach			
Model	Include *Z*	Stratify by *Z*	Residualize by *Z*	Ignore *Z*

1. ADDITIVE	+/+	+/+	+/+	+/+
2. ADDITIVE WITH CONFOUNDING	++/+	+/+	+/+	++/++
3. ADDITIVE WITH EFFECT MEDIATOR	- -/- -	- -/- -	- -/- -	-/-
4. INTERACTION WITH MAIN EFFECTS	++/++	++/++	++/++	++/++
5. INTERACTION WITH NO MAIN EFFECTS	+/++	+/++	-/+	-/+
6. CONDITIONAL ASSOCIATION	+/+	+/+	-/+	-/+

Summary of simulation results in Figures 1 and 2: Results are given in pairs corresponding to RF and MARS respectively; a "+" indicates reasonable TDR (≥ 80%) for detecting moderate effect sizes (≥ 0.5); "++" indicates reasonable TDR (≥ 80%) for detecting small effect sizes (≥ 0.3);"-" indicates lower TDR (50% to 80%) for moderate effect sizes; and '--" indicates very low TDR (< 20%) at moderate effect sizes. Correlation between *X*_1 _and *Z *is fixed at 0.5 for MODEL 2.

**Figure 3:**
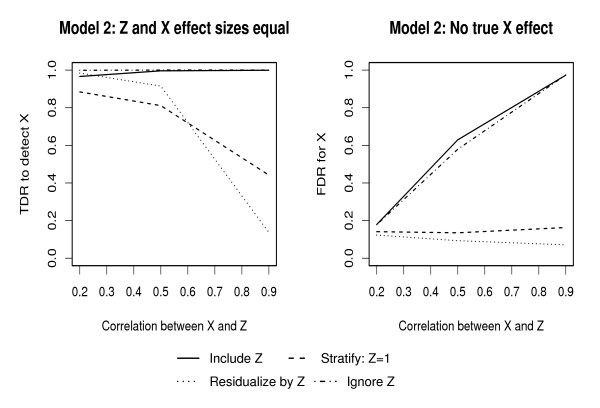
True and false discovery rates for genotype effects with correlated predictors using Random Forests: The first plot illustrates the effect of confounding on TDR when *X*_1 _and *Z *effects are fixed at 0.5, while the second plot illustrates FDR given that the *Z *effect is fixed at 0.5.

**Figure 4:**
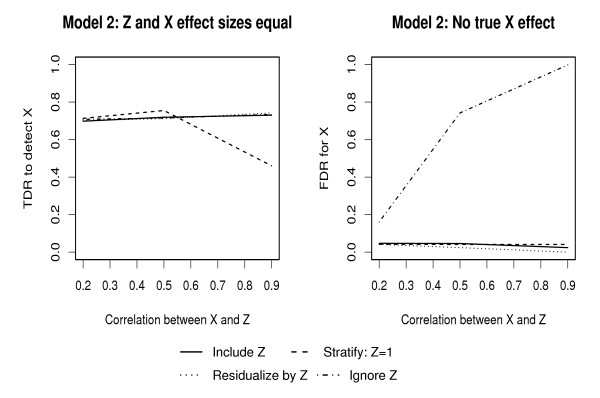
True and false discovery rates for genotype effects with correlated predictors using MARS: *Z *and *X*_1 _effect sizes are as in Figure 3.

Plots of changes in TDR and FDR under MODEL 2 with change is correlation between *X*_1 _and *Z *are given in Figures 3 and 4, for RF and MARS respectively. A summary of these figures is given in Table 2. Under RFs, there is high TDR to detect the genotype effect for all levels of correlation when the include and ignore approaches are used; however, these approaches also lead to high FDR with increasing levels of correlation between predictors. Stratifying and residualizing result in a reduction in TDR at levels of correlation ≥ 0.5, and consistently low FDR. Under MARS, including, residualizing and ignoring *Z *result in reasonable TDR to detect the genotype effect while stratifying results in reduction in TDR for correlation > 0.5. FDR under MARS is consistently < 10% when including, stratifying and residualizing but increases with level of correlation when the ignore approach is used.

**Table 2:** TDR and FDR under Model 2

	RF		MARS	
Approach	TDR	FDR	TDR	FDR

1. Include *Z*	+	-	+	+
2. Stratify by *Z*	-	+	-	+
3. Residualize by *Z*	-	+	+	+
4. Ignore *Z*	+	-	+	-

Correlation between *X*_1 _and *Z *is varied as shown in Figures 3 and 4: "+" indicates reasonable TDR (> 80 for RF and > 70% for MARS) or low FDR (< 20% for RF and < 10% for MARS) for all levels of correlation; and "-" indicates decreasing TDR or increasing FDR as correlation increases.

The simulations are carried out again with genotype frequencies equal to 0.25, and the results (not shown) are consistent with those for frequencies equal to 0.5, but with relatively lower TDR. Lastly, the simulations with a continuous *Z *are carried out for a fixed *X*_1 _and *Z *effect size of 0.5 and the results are given in Table 3. In this case, three of the four approaches to handling covariates are applied, namely, including, residualizing and ignoring covariates. For ease of interpretation, a summary of these results is given in Table 4. We look at the comparative performance of RF and MARS for this simulation for each model in turn. Under MODEL 1, we have reasonable TDR to detect genotype except when we include *Z *for MARS. In this case, TDR is relatively low at 55%. Under MODEL 2 with confounding, reasonable TDR is achieved only when include and residualize are applied under RF. Under MODEL 3, reasonable TDR is achieved when *Z *is included under RF; however we have lower TDR when ignoring Z and residualizing reduces TDR to < 20%. Under MODEL 4 both RF and MARS show reasonable TDR to detect the genotype in all cases. This is consistent with the result of binary *Z *under this model. Under MODELS 5 and 6 reasonable TDR is only achieved when Z is included for both RF and MARS. When applying these ML algorithms under the null model of no *X *or *Z *association with *Y*, given that *Z *is continuous, we obtain an FDR of 13% for RF and 19% for MARS.

**Table 3:** TDR for detecting genotype effects in the presence of a continuous covariate

	Approach		
MODEL	Include *Z*	Residualize by *Z*	Ignore *Z*

MODEL 1: ADDITIVE			
*β*/*σ *= *γ*/*σ *= 0.5	0.969/0.546	0.993/0.999	0.965/0.995

MODEL 2: ADDITIVE WITH CONFOUNDING			
*β*/*σ *= *γ*/*σ *= 0.5	0.922/0.729	0.942/0.731	0.116/0.740

MODEL 3: ADDITIVE WITH EFFECT MEDIATION			
*β*/*σ *= 0.5, *γ*/*σ *= 2	0.829/0.703	0.133/0.033	0.510/0.628

MODEL 4: INTERACTION			
*β*/*σ *= *γ*/*σ *= *ζ*/*σ *= 0.5	0.999/0.909	0.995/0.998	0.930/0.984

MODEL 5: INTERACTION, NO MAIN EFFECTS			
*ζ*/*σ *= 0.5	0.898/0.963	0.137/0.033	0.137/0.036

MODEL 6: CONDITIONAL ASSOCIATION			
*γ*/*σ *= *ζ*/*σ *= 0.5	0.906/0.854	0.119/0.037	0.127/0.045

TDR is given in pairs corresponding to RF and MARS, respectively.

**Table 4:** Summary of results in Table 3

MODEL	Include Z	Residualize by Z	Ignore Z
MODEL 1: ADDITIVE	+/-	+/+	+/+
MODEL 2: ADDITIVE WITH CONFOUNDING	+/-	+/-	- -/-
MODEL 3: ADDITIVE WITH EFFECT MEDIATION	+/-	- -/- -	-/-
MODEL 4: INTERACTION WITH MAIN EFFECT	+/+	+/+	+/+
MODEL 5: INTERACTION WITH NO MAIN EFFECT	+/+	- -/- -	- -/- -
MODEL 6: CONDITIONAL ASSOCIATION	+/+	- -/- -	- -/- -

Results are given in pairs corresponding to RF and MARS, respectively:

"+" denotes reasonable TDR (> 80%) to detect genotype;

"-" denotes lower TDR (50% – 80%) to detect genotype;

and "--" denotes very low TDR (< 50%) to detect genotype.

### HIV data example

We apply each approach to handling covariates using both RF and MARS on a genetics data set of HIV-1 infected individuals collected as part of the AIDS Clinical Trials Group (ACTG) New Works Concept Sheet 224 (NWCS224). The data set includes 626 individuals who are on combination anti-retroviral therapy (ARTs). We focus on the level of high-density lipoprotein cholesterol (HDL-c) as the trait (outcome) of interest, and consider 4 genes [Apolipoprotein C-III (ApoC-III), Apolipoprotein-E (ApoE), Endothelial Lipase(EL) and Hepatic Lipase (HL)] with 5, 2, 3 and 4 SNPs, respectively, as potential predictors. First stage analysis results, including complete demographic and clinical information on this cohort are presented in Foulkes, A. et al. (2006) [29]. An application of the RF algorithm to these data within the context of unobservable phase is presented in Nonyane, B. and Foulkes, A. (2007) [30].

Our analysis is limited to *n *= 512 individuals with a known duration of ART exposure and complete data in HDL-c. Among these are 317 Whites/non-Hispanic, 92 Blacks/non-Hispanic and 103 Hispanics. Some studies have shown that genetic variation plays a role in lipid differences across various races or ethnic groups [31-33]. For the ACTG data set, race/ethnicity was shown to be significantly predictive of plasma lipids changes and there is some evidence of race/ethnic differences in the association between lipid abnormalities and genotypes among patients on ARTs [29]. We therefore carry out the analysis with the expectation that RF and MARS may find a strong effect of race/ethnicity as well as some evidence of confounding or interaction between this covariate and genotypes in predicting HDL-c levels.

We begin by log transforming HDL-c and fitting a model with all 14 SNPs as well as race/ethnicity as potential predictors of log(HDL-c) levels. Secondly, we fit a model in which race/ethnicity is excluded from input. Thirdly, we fit a model after residualizing by race/ethnicity and lastly, we fit separate models for the three different ethnic groups. The results are given in Table 5 for RF and 6 for MARS. We run RF multiple times (*M *= 1000) to ensure stability [11]. Table 5 lists the median standardized variable importance score and standard error for each predictor. In the first approach, race/ethnicity is seen to have the highest median ranking above all predictors, followed by the ApoC-III [-482C/T (rs2854117)] SNP. Residualizing and ignoring this covariate give similar SNP rankings and results in the same ApoC-III SNP as the most important one. Stratifying by race suggests that the gene-HDL-c relationship is potentially modified by this covariate since different SNP subsets are ranked highest in the 3 racial groups. This represents an example that supports the simulation findings for MODELS 5 and 6 with interaction terms. Specifically, TDR is low for detecting SNPs ApoC-III [Gly34GlyC/T rs4520], EL [rs12970066] and HL [rs6084] unless a stratified analysis is performed. Notably, for the Whites/non-Hispanic group, which makes up the majority of the sample, the two most predictive SNPs match those that were selected using other approaches.

**Table 5:** Random Forest results of the analysis of ACTG data with HDL-c levels as trait and SNPs from ApoCIII, ApoE, EL and HL genes and race/ethnicity as predictors

	Include*	Ignore*	Residualize*	White (n = 317)	Stratify* Black (n = 92)	Hispanic (n = 103)
**ApoC-III**						
-482C/T (rs2854117)	**12.68**(0.63)	**11.81**(0.60)	**13.07**(0.66)	**13.05**(0.66)	-2.04(0.96)	1.19(0.97)
-455T/C (rs2854116)	9.04(0.66)	**9.69**(0.66)	**10.13**(0.67)	**8.90**(0.69)	-0.57(0.98)	1.27(0.98)
intron 1 (466)G/C (rs2070669)	4.70(0.93)	5.85(0.88)	5.91(0.90)	4.55(0.91)	-2.78(0.94)	-2.62(1.00)
Gly34Gly C/T (rs4520)	8.86(1.12)	5.51(1.03)	4.77(1.07)	2.99(1.02)	0.91(0.99)	**11.87**(0.91)
exon 4 SstI 4348(5) C/G(rs5128)	0.60(1.01)	2.12(1.04)	2.34(1.04)	2.91(1.03)	-3.20(0.97)	2.95(1.00)
						
**ApoE**						
Arg112Cys T/C (rs429358)	4.87(1.08)	1.45(1.00)	2.29(1.06)	6.29(1.08)	-5.22(0.96)	6.30(0.98)
Arg158Cys T/C (rs7412)	6.02(0.98)	7.49(1.00)	7.49(0.93)	5.08(0.92)	-5.20(0.97)	2.69(0.98)
						
**EL**						
rs12970066,	3.94(1.03)	4.00(0.97)	5.54(0.97)	1.12(0.97)	**8.57**(0.91)	-1.81(1.05)
Asn396Ser,	7.06(1.03)	8.30(1.05)	8.39(1.10)	2.27(1.04)	5.93(0.90)	2.09(0.97)
rs3829632 (-1309A/G)	-1.10 (0.99)	1.24(0.98)	2.25(1.04)	-1.64(0.98)	0.00(0.00)	-2.25(0.96)
						
**HL**						
rs2070895	**11.81**(1.04)	7.86 (1.09)	5.54(0.99)	5.58(1.11)	-1.73(0.95)	2.82(0.97)
rs12595191	-0.93(0.97)	-1.77(1.00)	-1.07(0.99)	-3.62(1.00)	-1.99(0.99)	0.07(0.99)
rs690	10.41(1.08)	1.28(0.99)	0.53(0.98)	3.93(1.05)	-3.98(0.97)	9.61(0.96)
rs6084	7.42(1.01)	6.47(1.01)	6.27(1.06)	-1.15(1.01)	**9.31**(0.86)	**10.97**(0.90)
						
Race/ethnicity	**21.58**(1.15)	NA	NA	NA	NA	NA

" * " indicates the approach to handling the race/ethnicity covariate;

"NA" indicates that the predictor was not included in the analysis.

The two highest importance scores from RF are in bold.

**Table 6:** MARS results of the analysis of ACTG data with HDL-c levels as trait and SNPs from ApoCIII, ApoE, EL and HL genes and race/ethnicity as predictors

	Include*	Ignore*	Residualize*	White (n = 317)	Stratify* Black (n = 92)	Hispanic (n = 103)
**ApoC-III**						
-482C/T (rs2854117)	4	-	1	1	-	-
-455T/C (rs2854116)	-	3	2	3	-	-
intron 1 (466)G/C (rs2070669)	-	-	-	-	-	-
Gly34Gly C/T (rs4520)	-	-	-	-	-	-
exon 4 SstI 4348(5) C/G (rs5128)	-	-	-	-	-	-
						
**ApoE**						
Arg112Cys T/C (rs429358)	-	-	1	-	-	-
Arg158Cys T/C (rs7412)	3	1	-	2	-	-
						
**EL**						
rs12970066,	5	-	-	4	1	-
Asn396Ser,	4	-	-	1	1	-
rs3829632 (-1309A/G)	-	-	-	-	-	-
						
**HL**						
rs2070895	3	1	-	2	-	-
rs12595191	-	-	-	-	-	-
rs690,	4	2	-	1	-	-
rs6084	2	-	1	-	1	-
						
Race/ethnicity	1	NA	NA	NA	NA	NA

" * " indicates the approach to handling the race/ethnicity covariate;

"NA" indicates that the predictor was not included in the analysis;

and "-" indicates that the predictor was not selected in the final MARS model.

Table 6 gives results of the analysis using MARS. Unlike RF, multiple runs of MARS on the same data set produce the same results and therefore we only run MARS once for this data set. For each of the predictors that were selected in the final model we report the order in which they were selected during the model-fitting process. When two or more predictors have the same ordering it implies that they act in interaction with each other at the corresponding model-fitting stage. Like in RF, race/ethnicity is selected as the most predictive of all predictors in the first approach. The SNPs selected using this approach differ from RFs. Interestingly, MARS identifies the HL [rs6084] SNP that RF only detected with the stratified analysis. This result is consistent with the simulation study findings that suggest MARS has a greater TDR for detecting genotype effects that interact with the covariate (MODELS 5 and 6) under the include approach. Interestingly, for the Hispanics group, none of the SNPs are selected under MARS, though several SNPs appear in the RF analysis. This may reflect the loss in power associated with subset analyses.

## Discussion

The aim of this investigation is to characterize the relative performances of RF and MARS algorithms for different models of association in genetic studies of unrelated individuals. Our study was motivated by the fact that even though there exists extensive genetic epidemiology literature about applications of ML algorithms in this setting, it is not well established how these algorithms perform under different approaches to handling covariates and various models of association. Overall, our investigation found that for a binary covariate: (1) machine learning algorithms perform relatively poorly in the context of effect mediation and the best approach in this setting is to ignore the mediator variable; (2) in the context of confounding, for high levels of correlation (> 0.5), it is best to use MARS and include the confounder or residualize. While TDR is lower for RF, MARS maintains control of FDR in these cases and is preferable; and (3) in the presence of interaction with no main effects and conditional association MARS performs better and it is best to include or stratify by the covariate.

The results of a continuous covariate vary and suggest that RFs perform better in most cases, though MARS and RFs are equally powerful when there is interaction with main effects. In a recent manuscript, Strobl, C. et al. (2007) [34] demonstrate that RFs can result in biased predictions in the case of both continuous and categorical predictors and they propose an alternative implementation of RF. In the analysis of the ACTG data set, both RF and MARS showed a strong association between HDL-c and race/ethnicity and evidence of underlying gene by race/ethnicity interaction as was shown in previous studies. MARS provides for uncovering gene by gene interaction both across and within racial groups.

Notably, machine learning algorithms are highly amenable to the analysis of a large number of input variables, as documented in the literature referenced in the introduction. We found that the computational burden of RFs using the randomForest package in R is greater than that of MARS, using the earth package which is also implemented in R. This is likely due to the fact that RFs involves fitting an ensemble of trees while MARS fits the equivalent of a single tree. This discrepancy will increase as the number of inputs increases. In this manuscript, we limit our simulation study to include 8 genotype indicators for the purpose of a coherent presentation. We also limit our analysis of the importance of genes and covariates to the relative rankings in RF and appearance in the final MARS model for ease of comparison between the two algorithms. A formal test of significance can be achieved using the robust inference approach described in van der Laan, M. (2006) [35]. Control for multiple testing is imperative for formal testing in this high-dimensional setting due to inflation of type-1 error, as evidenced in our simulation under the global null model in the Results section.

For the purpose of a concise presentation, we focus our approach and discussion on the evaluation of alternative strategies for incorporating covariates. Several important concepts require further consideration. Specifically, the simulation study focuses on a quantitative trait as the primary outcome of interest; and the impacts of genotyping errors, missing (genotype or covariate) data, variable penetrance and allelic heterogeneity are not considered. Extensive research supports the relevance of these factors on TDRs and FDRs. We further note that genetic association studies often involve a large number of SNPs. While our simulation study involves a small number of genotype predictors for clarity of presentation, ML algorithms have been widely applied to genetic studies of much higher dimensions [11,14,15]. Finally, we present our study in terms of gene-covariate interactions; however, our findings with regard to categorical covariates are equally applicable to gene-gene interactions that are coded similarly.

## Conclusion

In this manuscript we present the results of the study whose main aim was to compare approaches for handling the combination of SNPs and covariates within and across two ML algorithms. Based on the simulation study and the example, we conclude that both RFs and MARS are powerful algorithms for selecting subsets of significant predictors, but careful consideration of how to deal with underlying gene-covariate-trait relationships is important, particularly if confounding or effect mediation is likely. This study may be easily extended to other existing ML algorithms.

## Authors' contributions

BAS drafted the manuscript, assisted in the design of the simulation study, developed software and implemented the simulations, and analyzed the example data. ASF conceptualized the problem and assisted in design of the simulation studies and drafting the final manuscript.
